# Observational spatial memory in wolves and dogs

**DOI:** 10.1371/journal.pone.0290547

**Published:** 2023-09-13

**Authors:** Sebastian G. Vetter, Louise Rangheard, Lena Schaidl, Kurt Kotrschal, Friederike Range

**Affiliations:** 1 Domestication Lab, Konrad Lorenz Institute of Ethology, Department of Interdisciplinary Life Sciences, University of Veterinary Medicine Vienna, Wien, Austria; 2 Department of Behavioral and Cognitive Biology, University of Vienna, Wien, Austria; National University of Singapore, SINGAPORE

## Abstract

Social learning is highly adaptive in transmitting essential information between individuals in many species. While several mechanisms have been observed, less is known about how much animals can remember. However, results on observational spatial memory among caching species, i.e. a form of social learning allowing individuals to remember and pilfer food caches made by others, suggest that this ability correlates with their social organization. Both wolves and their domesticated form, dogs, are social species known to make food caches, and previous studies have shown that they both can use observational spatial memory abilities to find hidden food. In order to test how much socially transmitted information wolves and dogs can remember, we tested both species in a task requiring them to find 4, 6 or 8 caches after they observed a human hiding food items, or after a control condition where they could not observe the hiding. We found that both wolves and dogs retrieved more caches and were more efficient for the first few caches if they observed the hiding than in the control condition, suggesting that they did not simply rely on scent to find the rewards. Interestingly, wolves outperformed dogs irrespective of whether the caching could be observed or not. We suggest that this result is due to a difference in motivation/persistence between wolves and dogs rather than observational spatial memory.

## Introduction

Social learning, i.e. learning influenced by observation of or via interaction with another individual or its product [1], is highly adaptive and has been shown to play an important role in many species including birds, fishes, mammals, and marsupials with regard to food acquisition, predator recognition and avoidance, mate choice and intraspecific communication [2–5]. Several mechanisms of social learning have been observed across animal species [6, 7], however, the most common one might be stimulus enhancement (drawing attention to an object) and local enhancement (drawing attention to a location) [3, 6, 8].

One of the mechanisms involved is observational spatial memory (OSM: the ability to remember the location of food caches made by others after having observed the hiding process; [9]. Such, OSM involves delayed local enhancement, and object permanence skills (memory for items that are temporarily out of view; [9]. Studies on OSM in the context of pilfering behaviour in corvid birds such as pinyon jays (*Gymnorhinus cyanocephalus*), mexican jays (*Aphelocoma ultramarine*), clark’s nutcracker, (*Nucifraga columbiana*), jackdaws (*Corvus monedula*) and ravens (*Corvus corax*) [9–12], show that the ability to remember socially learned information varies quantitatively between species (see also [13] for a review on food caching corvid’s behavior and cognitive abilities). Grodzinski & Clayton [13] suggest that such differences in OSM may depend on both the social structure and the frequency of witnessing caching events. Indeed, highly social and caching species such as pinyon jays, mexican jays, ravens and western scrub-jays (*Aphelocoma californica*) perform particularly well at pilfering caches, while the rather solitary but caching clark’s nutcrackers and the highly social but non-caching Jackdaws are less efficient pilferers. Among the canids, coyotes (*Canis laterans*) [14], red foxes (*Vulpes vulpes*) [15, 16], arctic foxes (*Alopex lagopus*) [17], african wild dogs (*Lycaon pictus*) [18], and wolves [19–21] are known to make food caches. For example, wolves hunt large prey, which, because it often cannot be consumed at once, can result in a surplus of food [22]. Accordingly, wolves in the wild and in captivity have been repeatedly observed to make food caches [20, 23], consisting of approximately 0.65 kg of regurgitated food, parts of carcasses [20, 21], and even whole carcasses of 6 to 8 kg [24]. Pups in the wild have also been observed to cache food around the den when a surplus of food was available [25]. Thus, although most records are anecdotal, food caching seems to occur regularly [20, 22] and may be more important in summer than winter, i.e., when wolves forage alone or in small groups [22]. Similarly, pilfering of caches by pack-mates has been observed in the wild (David Mech, personal communication in [26]). The frequency of pilfering may affect the complexity of the food caching strategy as demonstrated in corvids [27]. A previous experimental study revealed that wolves do pay attention when social partners hide food, and may retrieve it later [26]. However, it is unknown how many caches they could potentially remember, and little is known about the frequency of pilfering in wolves.

Similarly, dogs, the domesticated form of wolves, also cache and retrieve food items. For example, according to Miklósi [28], Grzimek [29] and Heimburger [30] show that dogs can remember a disappeared food item for up to 30 minutes. In addition, citing Beritashvili [31], Miklósi [28] further states that, even though the study could have had better controlled conditions, dogs remembered the location of a food item the day after it has been hidden. Moreover, similarly to wolves, dogs retrieved food items hidden by a conspecific or human partner after observing the hiding process [3, 26, 32]. Interestingly, the experiment by Range & Virányi [26], which was conducted with similarly raised wolves and dogs and therefore specifically aimed at the possible effect of domestication on social learning, found no differences between the two species [26]. However, while both may be equally good in using simple social learning mechanisms, their abilities might deviate if the cognitive demand of the task increases, e.g., by having to remember and retrieve not just one, but several food items.

Due to a change in the ecology of dogs during domestication, where humans ease access to food, buffering environmental effects, natural selection for food-related problem solving may have been relaxed [33, 34] suggesting better cognitive performance of wolves in food-related tasks. To test this hypothesis, we investigated potential quantitative differences of OSM abilities in wolves and dogs in a task cognitively more demanding than the one we used previously, where we did not find any differences between the two species [26]. Across several trials, the animals observed a demonstrator hiding a varying number of food items (4, 6 or 8), which the animals were allowed to retrieve afterwards within a certain period of time. Because wolves and dogs have shown OSM abilities in previous studies [26, 28], we predicted them to be more successful and more efficient in terms of latency and distance travelled to find the caches after witnessing the caching compared with the control condition, in which–all else being equal—the hiding could not be observed and thus could only be solved by other means (e.g. odour, individual searching strategies). No differences between test and control conditions would indicate that the animals do not use OSM to find the food. Finally, based on the domestication hypothesis outlined above and the fact that the local enhancement task was increased in difficulty, we predicted wolves to outperform dogs.

## Materials and methods

No special permission is required in Austria for using animals in such cognitive studies. The applicatory committee for research without special permission regarding animals is the ‘Tierversuchskommission am Bundesministerium für Bildung, Wissenschaft und Forschung (Austria)’.

### Subjects

The study was conducted with 9 timber wolves and 8 mongrel dogs, all hand raised and living at the Wolf Science Center in Ernsbrunn, Austria. Six of the wolves and all of the dogs had participated in the previous local enhancement study when 4 to 7 months of age. At the time of the study, both wolves and dogs lived in 4 packs in separate enclosures (wolves: 2x 8000 m², 1x 4000 m² and 1x 3000 m²; dogs: 4000 to 5000 m²), and were daily trained using positive reinforcement. They were used to participating in cognitive tests and to being separated from their pack for tests, training and leash walks. Wolves were fed two to three times a week with rabbits, chunks of meat, dry food, sausage or bones, while dogs were fed everyday between 17h00 and 18h00 with dry food. Water was provided *ad libitum*.

Experiments for this study were conducted over three periods. Three wolves, 17 to 20 months old at the time of the study, were tested between October and December 2009 and six wolves, 12 to 47 months old, were tested between April and August 2013. Eight dogs were tested between January and July 2014 and were between two and four years old when the experiment started. A detailed summary of age, sex, and genetic relationship of the tested animals can be seen in Table 1. The wolves have been repeatedly observed making food caches after feeding events. Cached items consisted of parts of rabbit or chicken (personal communication, Rita Takacs, February 2013), whole chicks or parts of a deer (personal observation, Lena Schaidl, August 2013).

**Table 1:** List of animals showing species, genetic relationship, sex (f = female, m = male), age, origin, pack structure and year of experiment.

Individual	Sex	Born	Origin	Sibling	Pack	Experiment
Wolves						
Tala	f	2012	Minnesota Wildlife Connection	Amarok	1	2013
Chitto	m	2012	Minnesota Wildlife Connection	Una	1	2013
Una	f	2012	Minnesota Wildlife Connection	Chitto	2	2013
Kenai	m	2010	Parc Safari, Kanada	-	3	2013
Geronimo	m	2009	Triple D Farm, Montana, USA	Yukon	3	2013
Yukon	f	2009	Triple D Farm, Montana, USA	Geronimo	2	2013
Shima	f	2008	Zoo Herberstein, Austria	Aragorn	4	2009
Aragorn	m	2008	Zoo Herberstein, Austria	Shima	4	2009
Kaspar	m	2008	Zoo Herberstein, Austria	-	4	2009
**Dogs**						
Bora	f	08/2011	Györ, Hungary	Layla	5	2014
Binti	f	09/2010	Siofok, Hungary	-	6	2014
Maisha	m	12/2009	Paks, Hungary	-	6	2014
Layla	f	08/2011	Györ, Hungary	Bora	7	2014
Nuru	m	06/2011	Paks, Hungary	Zuri	7	2014
Zuri	f	06/2011	Paks, Hungary	Nuru	7	2014
Meru	m	10/2010	Velence, Hungary	-	8	2014
Nia	f	07/2011	Paks, Hungary	-	8	2014

### Experimental site

**Fig 1:**
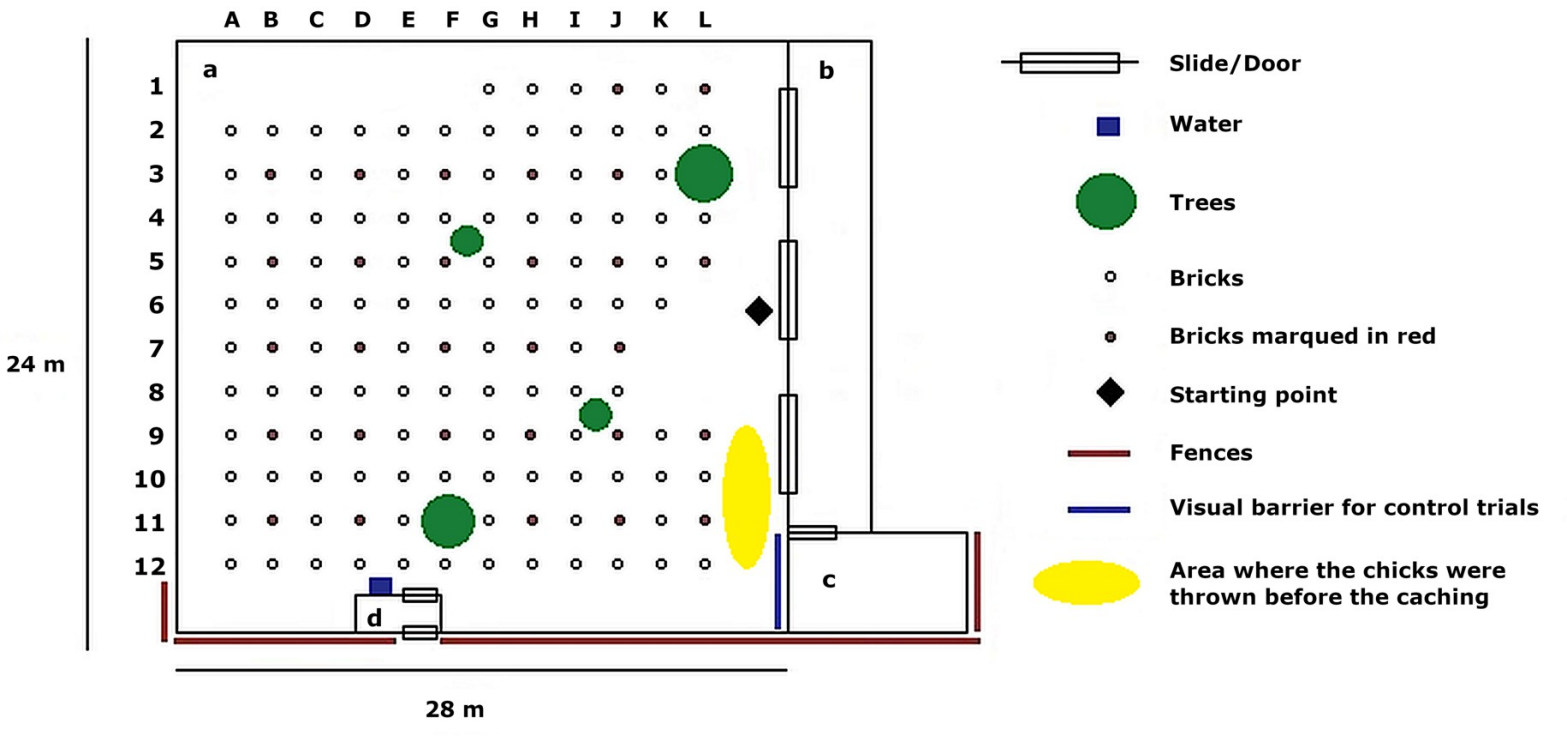
Outline of experimental site of 2013/2014. a) Testing enclosure; b) Shifting system; c) Compartment of the shifting system used during control trials; d) 2-door entrance for humans.

Experiments were conducted in a testing enclosure containing 131 bricks. Every brick represented a possible cache location. The testing enclosure of the year 2009 measured approximately 41 x 26 m and the test enclosure of 2013 and 2014 measured 28 x 25 m (Fig 1). The bricks were set up in a 2 x 2 m grid. In both test enclosures, the grids used for the set-up of the bricks measured about 670 m^2^, so that the conditions were comparable between 2009 and 2013/2014. The animals could watch the caching process (experimental condition) from a shifting system next to the test enclosure (area b, Fig 1), allowing them to move along the fence and see all parts of the enclosure.

### Experimental set up

The testing enclosure was located between two wolf enclosures and both dogs and wolves knew it from other cognitive tests. Opaque covers were fixed on the fences along the testing enclosure to ensure that the wolves living around it were not able to observe the caching process (Fig 1). This measure should prevent observational learning effects and distraction. As food items, we used whole one day old chicks (approx. 7–9 cm long) for wolves and half chicks (bottom part from under the wings) for dogs. As the dogs were smaller than the wolves, this should keep their motivation to retrieve the food and minimize the time spent eating during the experiment. Both wolves and dogs were used to this food reward from other experiments and showed high motivation to find them [26]. The animals had access to water ad libitum during the tests.

### Training sessions

Prior to testing, all animals had 16 training sessions. In each training, two items were hidden using two different “minimal retrieval distances” (i.e., the minimal distance the animal needs to travel to find the two items). Eight had a minimal retrieval distance of 20 m, the other eight of 40 m. Caches were made randomly in the order 1–2 or 2–1 (1–2 being the order matching the minimal retrieval distance). On each training day, the animals were trained once in both minimal retrieval distances, respectively. Hereby, the sequence of the training with respect to the two different minimal retrieval distances and the testing order of the animals was alternated randomly. The animal stayed in the tunnel system before the 2nd training session began. In case an animal did not find all the caches in a training session, the experimenter entered the testing enclosure, showed and fed the missed caches to the animal in order to increase its motivation to pay attention to the hiding process.

### Experimental sessions

During experimental sessions the individuals were offered the opportunity to retrieve caches either after they were able to observe the caching process (TEST) or after they were not able to observe the caching process (CONTROL). The control condition was carried out to test whether animals use odour cues to find the food, in which case we would not have expected a difference between the test and control conditions. To further investigate the differences in OSM, we varied the number of caches between 4, 6 and 8, with a minimal retrieval distance of 40 m, 60 m and 80 m (+/- 0.5 m), respectively. In each of the six possible settings (test or control trial with 4, 6 or 8 caches, respectively) each focal animal was tested in six separate trials (i.e., six repeats per animals and setting), resulting in a total of 36 experimental sessions per animal. All animals were tested at maximum in a single experimental session per day, and condition (i.e., test vs. control) and cache number were randomized within and between individuals to control for learning effects. Experimental sessions were on average 4 days (range 1–39 days) apart for dogs, 5 days (range 1–27 days) apart for wolves.

A program written in R 2.10.0 [35] randomly determined the cache locations. It allowed for setting the minimal retrieval distance, the minimal distance to obstacles (set to 0 m), the minimal distance from the starting point (set to 2 m), as well as a minimal distance between caches (set to 4 m, so there is always at least one grid-brick between two caches locations). The items were hidden in a different order from the one given by the program, which was the order the animal had to follow to retrieve the cache with the minimal retrieval distance. The caching order was set as following: 51637284 (8 caches), 415263 (6 caches) and 3142 for the 4-caches situation. The cache numbers hereby refer to their respective location regarding to the minimal retrieval distance as given by the program with “1” being the cache closest to the entrance for the animal.

### Experimental procedure

In both test and control condition, the focal animal was called into the shifting system (b, Fig 1), and the experimenter (2009: SV, 2013: LS, 2014: LR) threw the number of chicks to be cached one by one in front of the animal on the other side of the fence (Fig 1). The experimenter then entered the test enclosure and picked up the chicks one by one, while the animal was watching. This procedure was used to increase the motivation of the animal to follow the hiding process (test) and to ensure that the animal expects to find caches in the enclosure in control sessions.

In test trials, the experimenter made the caches while the subject could observe the caching process from the shifting system (b, Fig 1). In control trials, the animal was led into a part of the tunnel (c, Fig 1), where it could not watch the caching process due to a visual barrier. Because the animal could have still heard the experimenter walking around in the test enclosure while making caches, the caches were already made before the beginning of the test procedure. The experimenter sat still for two minutes and hid the chicks in a bag, while the animal was in the confined part of the tunnel system. The experimenter then left the test enclosure, while the animal was released back into the part of the tunnel system without a visual barrier.

In test trials, the animal was allowed to enter the test enclosure through the middle slide (Fig 1) and search for the caches one minute after the experimenter left the testing enclosure. In control trials, because the animal was not in visual contact with the chicks for two minutes and to ensure it remained motivated to search, it was released in the enclosure as soon as the experimenter left. This procedure also ensured that the animals had not seen a chick for approximately the same time duration in test and control trials. All animals had three minutes to find a cache. Every time a cache was found, the timer was again set to three minutes. The trial ended if the subject did not find another cache for three minutes, or three minutes after the last cache was recovered. After the trial ended, the animal was called back into the shifting system. Unrecovered caches were not shown to the animal but removed after the experiment to prevent further learning effects. Cache retrieval was recorded with a Sony HD R-C320E.

### Coding

The videos were coded using Solomon Coder version beta 14.05.19 (© 2014 by András Péter, ELTE TTK, Hungary). The time of searching a cache, handling and eating the cache, and being not interest in the caches (NIC) was coded for each retrieved cache separately and for three minutes after the last one was found and defined as follows:

- Searching: Walking, trotting or running in the grid and investigating a stone (standing and sniffing it less than three seconds).
- Handling: Manipulating or eating the recovered cache, and sniffing the cache location just after eating it.
- NIC: Neither eating nor looking for caches (e.g., looking outside the testing enclosure, investigating something else than a stone, sitting in the shade). If an animal spent more than three seconds sniffing a grid stone this was also considered as NIC.

For each video, the name of the dog or wolf, the trial condition, the number of caches, the temperature, the time of the day (morning: before 12h00; midday: from 12h00 until 16h00; afternoon: after 16h00), the weather and the soil condition (dry or humid) were recorded.

### Statistical analyses

Three measures were extracted from the videos to analyse the animals’ capability of remembering observed food caches: the number of unrecovered caches as an inverse measure of cache retrieval success, the latency of retrieving each successfully recovered cache, and the distance walked to retrieve each successfully recovered cache. The number of unrecovered caches was analysed in a mixed-effects Poisson model (package lme4) [36]. The latency and the distance travelled to retrieve each successfully recovered cache were analysed in two separate linear mixed effects models (package nlme) [37]. All three models contained the independent variables species, condition (i.e., test vs. control), trial number, their respective pairwise interaction with the number of caches made as well as the pairwise interaction between species and condition. Models on latency and distance travelled to retrieve single caches further contained the retrieval order of recovered caches as well as its interaction with the condition. Further, all three models additionally included the random effect of focal individual to correct for multiple testing. Full models were analysed regarding overdispersion, in the case of the Poisson model, and deviation from normality using histograms and qq-plots in the case of linear mixed effects models. The Poisson model showed no sign for overdispersion. Both linear models showed deviations from normality and were boxcox-transformed [38]. Both boxcox-transformed models showed no deviations from normality anymore. For all three models the respective best model was determined in a model selection table (package MuMIn) [39] based on the AICc (Akaike’s information criterion [40] corrected for small sample size [41]. All statistical analyses were performed in R 4.2.2 [42].

## Results

### Success in finding caches

**Fig 2:**
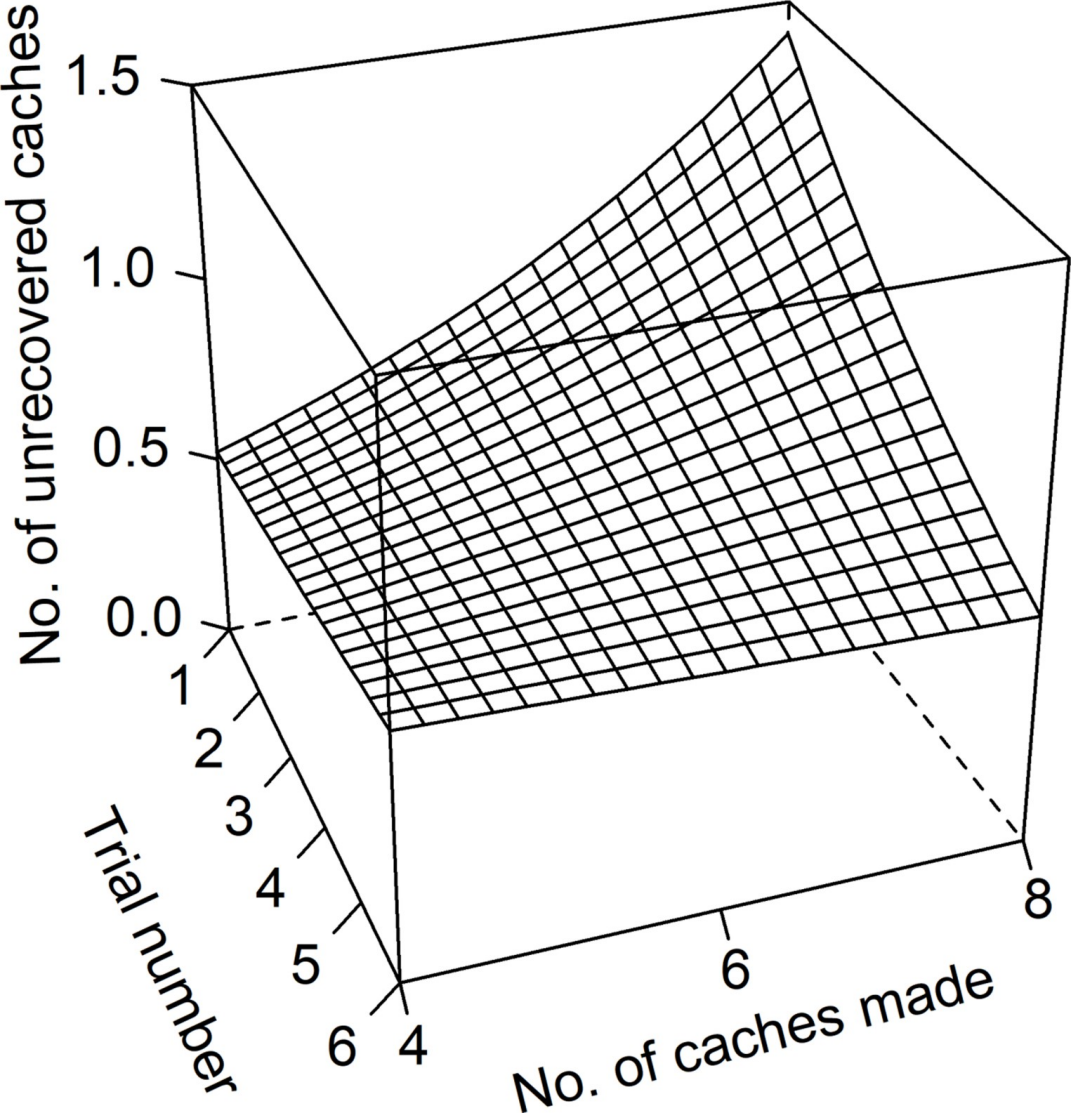
Partial effects plot of the interactive effect of the number of caches made and the trial number (i.e., number of repeats performed in a certain experimental setting) on the number of unrecovered caches.

Wolves and dogs were more successful in retrieving cached food after observing the hiding (estimate ± sd = -0.45 ± 0.09, z = -4.89, p < 0.001). Irrespective of condition (i.e., test vs. control), wolves were more successful than dogs in finding caches (estimate ± sd = -0.36 ± 0.11, z = -3.29, p = 0.001). Furthermore, there was a significant interaction between trial number and number of caches made (estimate ± sd = -0.05 ± 0.02, z = -3.29, p = 0.001; Fig 2). This interaction indicates that the animals’ success in retrieving caches was rather high and constant over the trials when only few caches were made (Fig 2). The more caches were made, the worse was the initial retrieval success which, however, improved with increasing trial numbers to levels comparable with the initial success for the low number of caches (estimate ± sd = -0.05 ± 0.02, z = -3.29, p = 0.001; Fig 2).

### Latency to find each successfully retrieved cache

**Fig 3:**
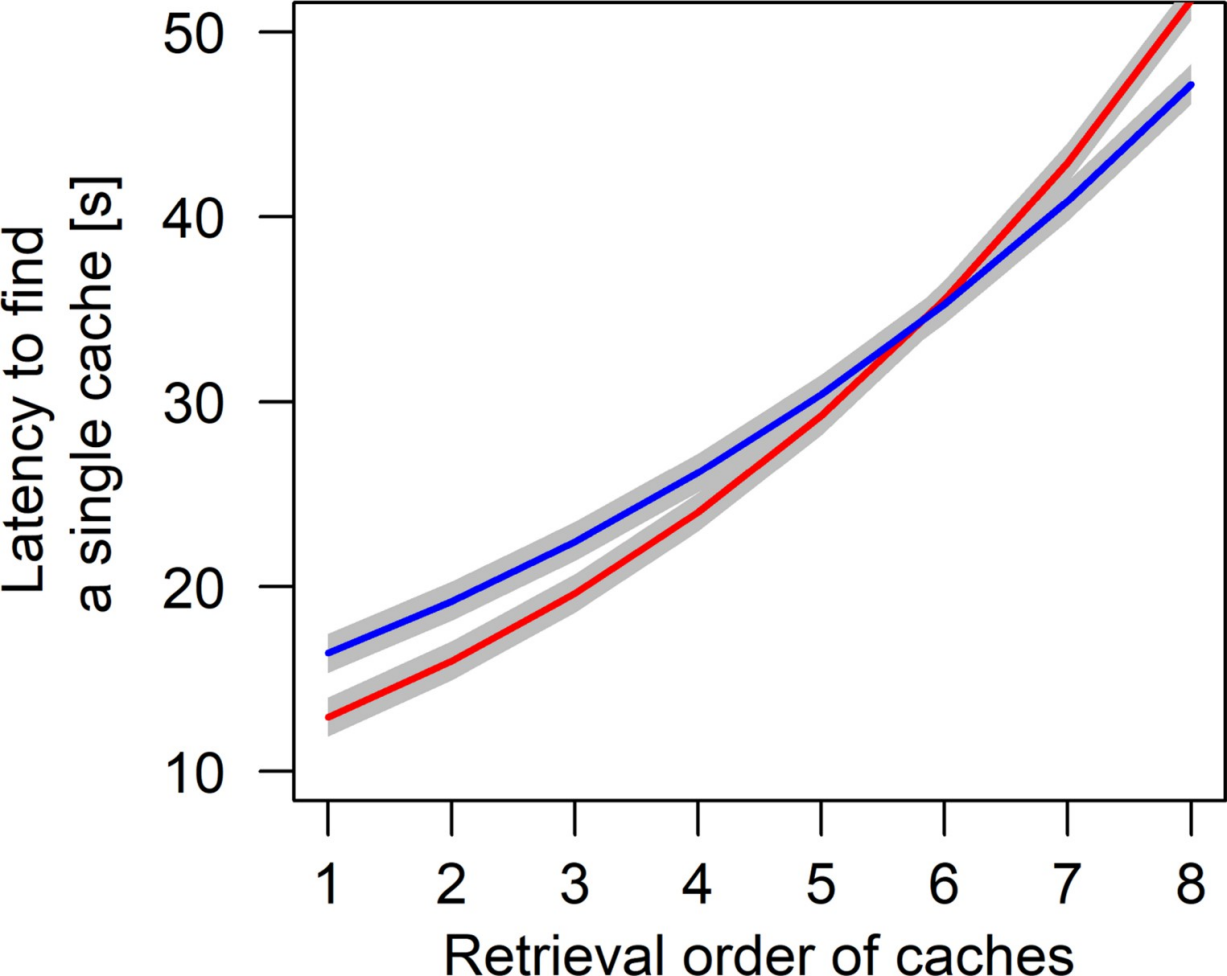
Partial effects plot of the interactive effect of condition and retrieval order of successfully recovered caches on the latency to retrieve a single cache (red = test, blue = control). Grey areas show standard errors of the regression line.

**Fig 4:**
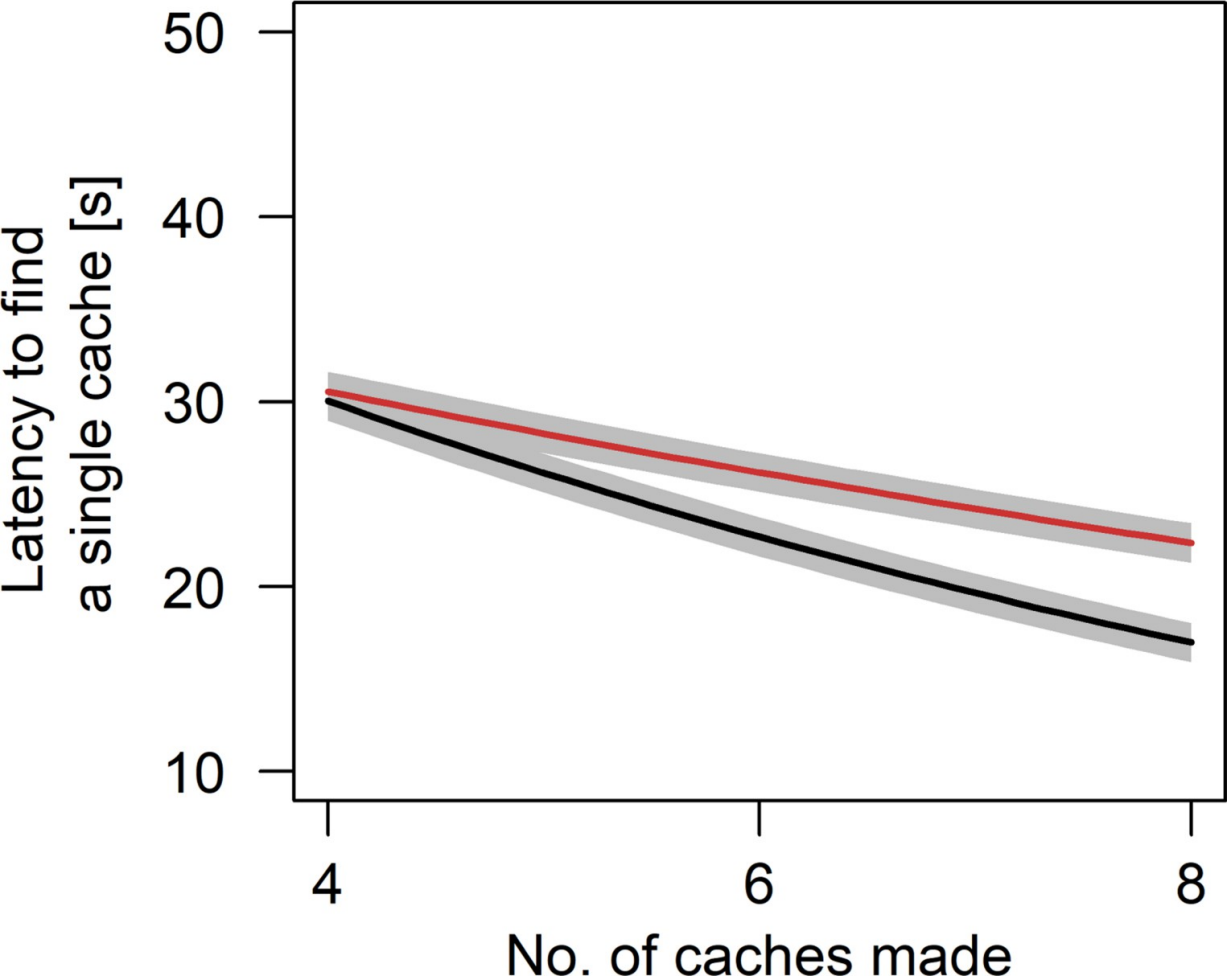
Partial effects plot of the interactive effect of species and the number of caches made on the latency to retrieve a single cache (brown = dogs, black = wolves). Grey areas show standard errors of the regression line.

**Fig 5:**
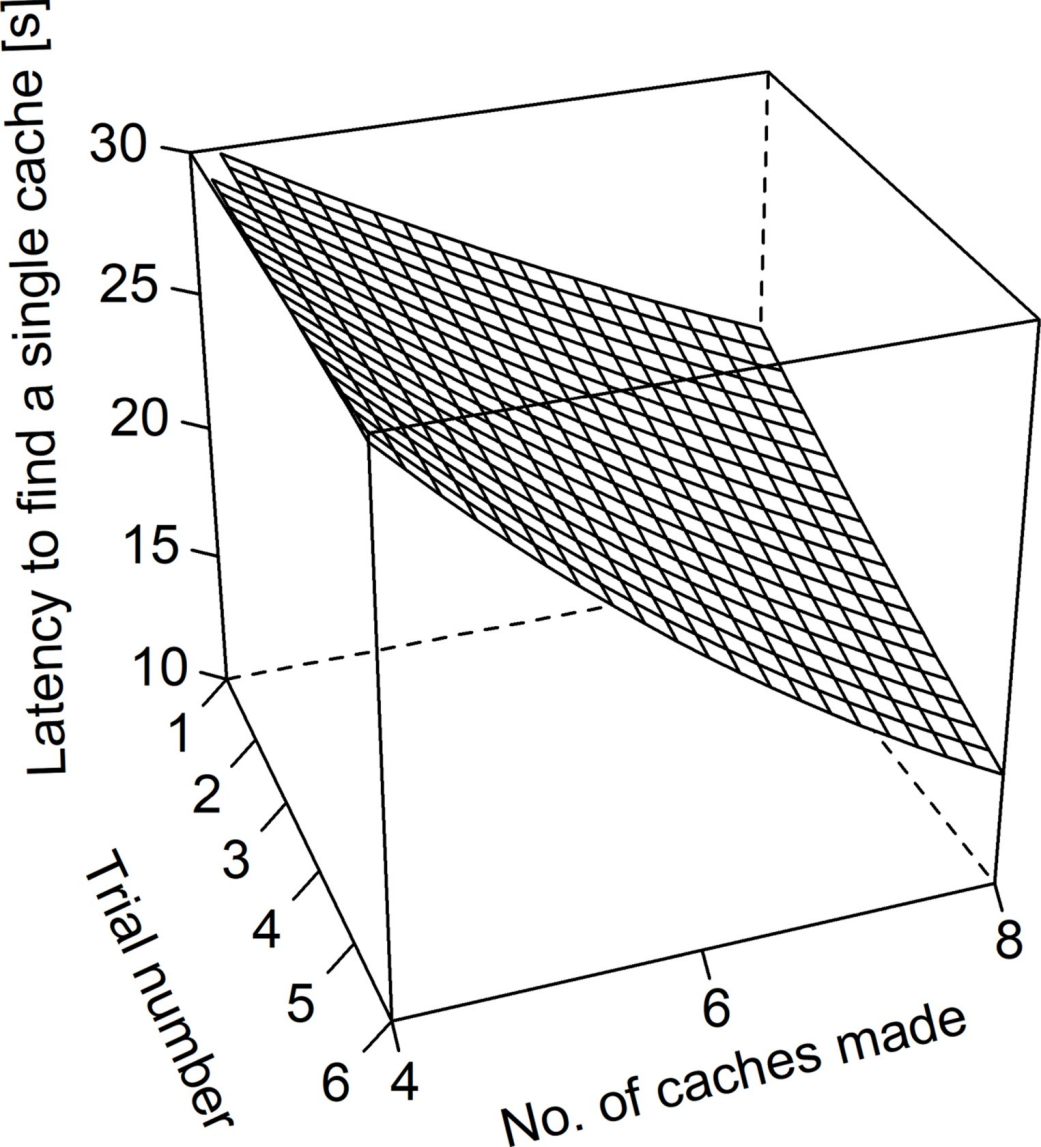
Partial effects plot of the interactive effect of the number of caches made and the trial number (i.e., number of repeats performed in a certain experimental setting) on the latency to retrieve a single cache.

Wolves and dogs retrieved the first four to five food caches faster after observing the hiding process than in the control condition. However, this effect disappeared for the 6^th^ to 8^th^ cache (estimate ± sd = 0.06 ± 0.02, t = 3.11, p = 0.002; Fig 3). Average latency to retrieve a single cache decreased with increasing number of caches made. This effect was stronger for wolves than for dogs, as shown by the interaction between species and the number of caches made (estimate ± sd = -0.09 ± 0.02, t = 3.49, p < 0.001; Fig 4). Further, there was an interaction between the trial number and the number of hidden caches (estimate ± sd = -0.02 ± 0.01, t = -3.04, p = 0.002; Fig 5). This effect indicates that the average latency to find a single cache decreased with an increasing number of caches made, with this effect being even more pronounced at the end of the experiment. In other words, the latency to retrieve each cache remained constant over trials with few caches. However, if more caches (6 or 8) were made the latency to retrieve a cache decreased over trials (Fig 5). Finally, there was a trend for an interaction of species and condition included in the best model suggesting that dogs generally needed longer to retrieve a single cache but benefitted more from observing the caching process than wolves (estimate ± sd = 0.14 ± 0.08, t = 1.85, p = 0.065).

### Distance travelled to find each successfully retrieved cache

**Fig 6:**
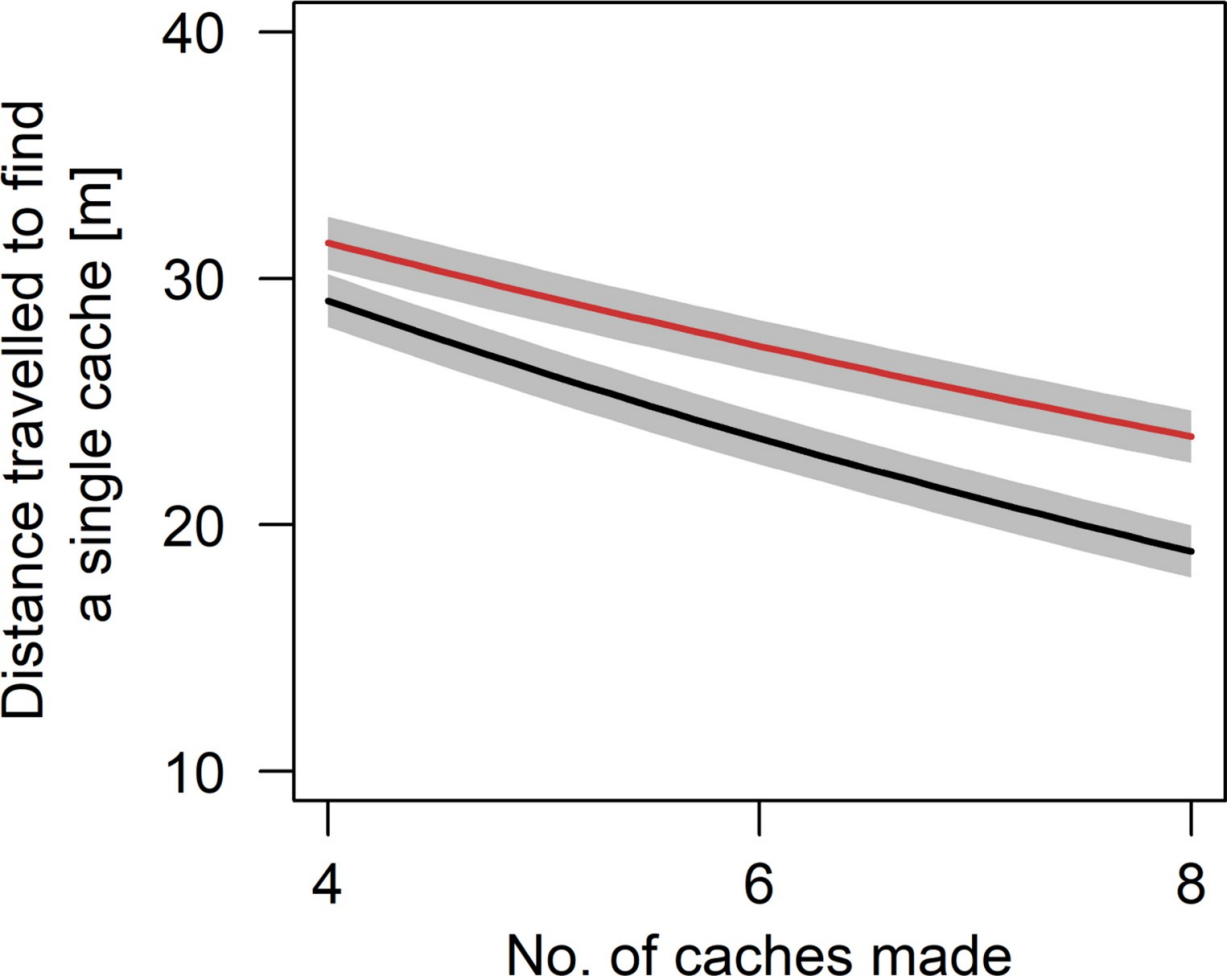
Partial effects plot of the interactive effect of species and the number of caches made on the distance travelled to retrieve a single cache (brown = dogs, black = wolves). Grey areas show standard errors of the regression line.

**Fig 7:**
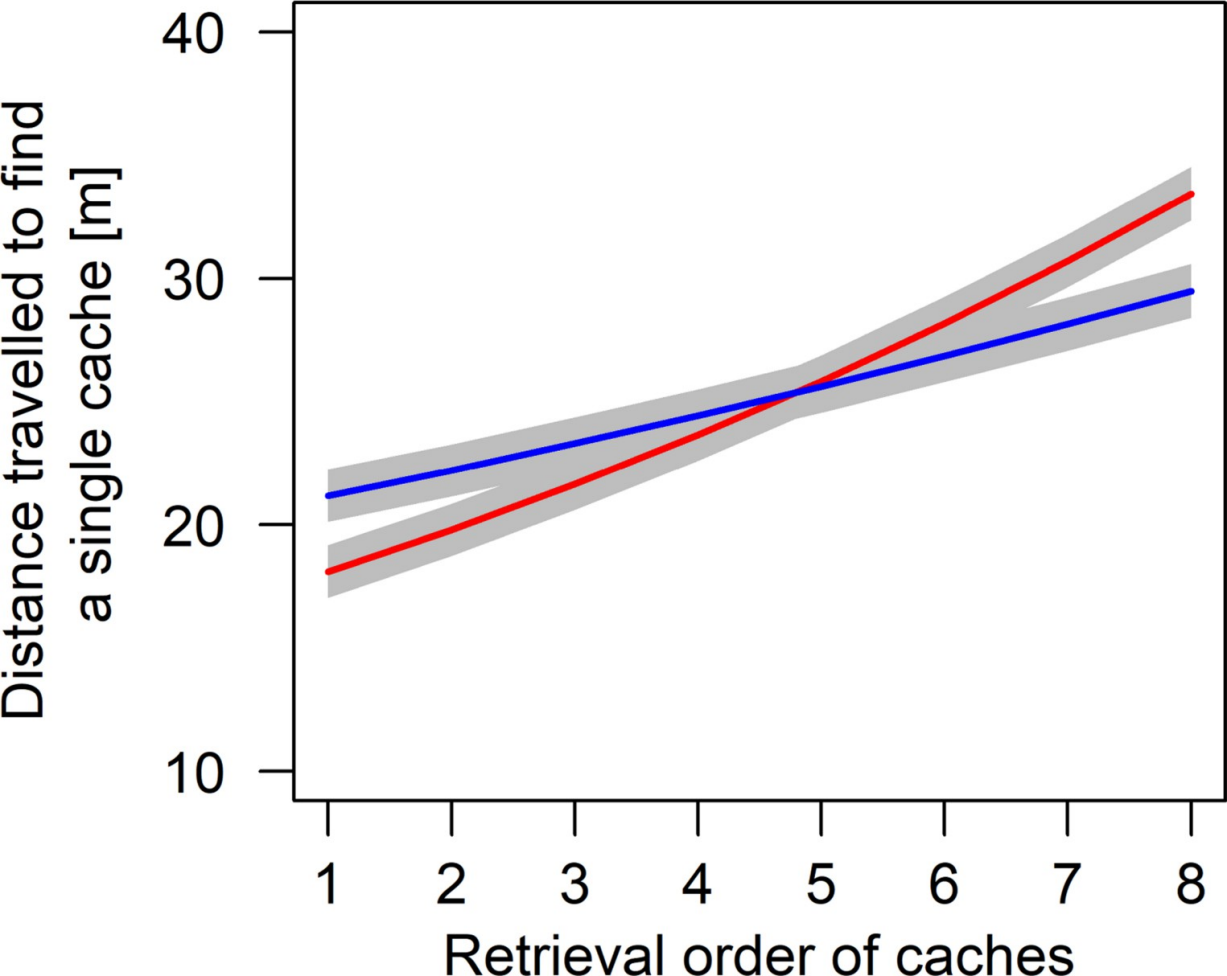
Partial effects plot of the interactive effect of condition and the retrieval order on the distance travelled to retrieve a single cache (red = test, blue = control). Grey areas show standard errors of the regression line.

The average distance travelled to retrieve a single cache decreased with increasing trial number (estimate ± sd = -0.05 ± 0.01, t = -4.71, p < 0.001). The interactive effect of the number of caches made and species showed that the average distance travelled to retrieve a single cache decreased with increasing number of hidden caches, and that this effect was stronger in wolves than in dogs (estimate ± sd = -0.05 ± 0.02, t = -1.99, p = 0.047; Fig 6). Further, there was a significant interactive effect showing that wolves and dogs retrieved the first few food caches with walking shorter distances after observing the hiding process than in the control condition. Similar to the latency above, however, this effect disappeared from the 4^th^ to 5^th^ recovered cache on (estimate ± sd = 0.06 ± 0.02, t = 2.82, p < 0.005; Fig 7).

## Discussion

### Differences between tests and controls

As predicted, both wolves and dogs not only left fewer caches unrecovered but were also faster and walked shorter distances to recover a cache in test conditions where they could observe the caching process as compared with the control condition where they could not. The latter, however, was only true for the first three to four caches recovered indicating that OSM in both species is limited and that with later caches they relied on odour cues or/and random search. Moreover, their success in retrieving caches increased over the course of the experiment, indicating a learning effect, e.g., in terms of search strategy (see also below), resulting in animals being as successful in finding eight caches towards the end of the experiment as finding four caches in the beginning. While wolves generally outperformed dogs, they did so in both the test and control conditions, suggesting that some other factors aside of mere OSM abilities were responsible for this difference.

The results that both wolves and dogs benefitted from the demonstration of the hiding process are in line with those of Range & Virányi [26], who showed that both species were significantly better to find hidden food if able to observe a conspecific or a human hiding it. Although it is clear that the animals benefited from the demonstrations, we cannot conclude that all food items found in the test sessions were remembered from the hiding process as also indicated by the fact that later caches were not recovered faster in the test than control trials. Indeed, while canids seem to rely preferably on visuals cues when those are provided (e.g., see [43] for a study on red foxes, and [44] for studies on coyotes), they do have a well-developed sense of smell. That they had 3 minutes to retrieve each cache and there was no punishment for looking at the wrong places, likely led the animals to combine visual and olfactory cues to find the food items in our task, especially if remembering became more demanding (e.g. with later caches). The fact that we found high retrieval rates in the control trials support the conclusion that also odour cues were used to find the caches. Similar results were found in the previous study of Range & Virányi [26].

Indeed, using a multi-cue system involving both memory and visual/olfactory cues while searching for hidden food has been proven to be more effective than relying only on memory. For example, yellow pine chipmunks (*Neotamias amoenus*) outperform steller’s jay (*Caynocitta stelleri*), because they also use olfaction to relocate caches [45].

Alternatively, the animals might have been just more motivated to search for the food (and thus were more successful) after witnessing the hiding process and seeing the food items for a longer time, but did not rely on any visual information to find the caches. However, both wolves and dogs were not only more successful in retrieving caches after observing the hiding process, they were also faster and walked shorter distances to recover the first three to four food items, suggesting that they remembered at least some of the cache locations and used OSM abilities to a certain extent.

Not surprisingly, the wolves and the dogs improved their efficiency in retrieving caches over the course of the experiment. While they were rather good at finding four caches from the beginning on, they achieved a similar success rate with the eight caches in the later trials with increasing experience. Moreover, both their latency and distance travelled to find a single cache decreased over trials. These results show that both wolves and dogs remained motivated to look for caches throughout the experiment, and indicate that over time, they developed a better search strategy.

Interestingly, their efficiency (time and distance travelled) improved not only in the test sessions, but also in control sessions. One possible explanation for this is that the animals learned that the number of chicks presented in the beginning of the session indicated the number of chicks hidden. However, given their numerical competence in such serial presentation of quantities seems limited at least in the dogs [46, 47], this is rather unlikely. We rather propose that the motivation to search increased throughout the experiment, possibly increasing the speed the animals were searching and/or optimizing the search strategy. Due to the size of the caching area and the optimal retrieval distance set up to retrieve the caches (40 m for 4 caches, 60 m for 6 and 80 m for 8 caches), the more caches were made, the more they tended to be on the bricks next to the fences of the enclosure. In other words, in the eight-cache situation, walking along the fences would be the best solution in terms of retrieval distance. Thus, animals might have simply learned over sessions that they should search in a certain area of the enclosure (along the fences), instead of remembering each cache or searching randomly.

### Differences between wolves and dogs

In both tests and controls, wolves outperformed dogs in their retrieval success, were faster in finding the caches and walked shorter distances to recover the caches. Since this effect was not specific to the test condition, differences in OSM between these two species cannot account for this result or only to some degree, supporting the hypothesis that these abilities are ancestral and were not influenced by the process of domestication. This result is in line with the previous, cognitively less demanding, study, where we also found no differences between wolves and dogs in a local enhancement task when only one chick was hidden [26]. The greater success of wolves might be explained by wolves having a better sense of smell than dogs and thus being able to find more caches. However, in a study testing the olfactory detection ability of dogs and wolves, Polgár et al. [48] found that except for the most difficult level (where only wolves and “scent breed” of dogs performed better than chance), all animals had a very high level of success. According to this, it seems unlikely that dogs and wolves differ in their ability to find the caches using olfactory cues in our setting (where the chicks were exposed). Along the same line, it is also possible, that, since we only used ½ chicks for the dogs, but whole chicks for the wolves that the whole carcass produced more smell than the half one and thus, facilitating the search for the wolves. However, we do not think that this is the case, but rather, if at all, the opposite is true: By cutting the little carcasses in half, we exposed the meat and intestines and the half carcasses actually smelled stronger than the whole carcasses. Thus, it is unlikely that olfactory cues or abilities are responsible for the differences observed. Another possible explanation is that wolves have a better search strategy than dogs. However, if this would have been the case, we would have also expected that wolves have a reduced travel distance, which was not the case. Finally, the wolves might have been more motivated by the food rewards we used (chicks) and thus searched longer and faster, increasing the number of caches found as well as reducing their latency to find them.

Wolves have been shown to be more persistent in several tasks than dogs, which probably can be explained by their respective ecological niche with wolves usually hunting, while free-ranging dogs usually feed on human refuse [49, 50]. Thus, selection for persistence could have been relaxed during the domestication process in dogs, or even selected *against* by humans [49].

Limitations of the study include the small sample size and, arguably, that most of the animals already participated in a local enhancement study in their first year of live questioning the generalizability of the results. While unfortunately it is not possible to increase the sample size in this setting due to the enormous costs involved when raising and keeping these animals and preparing them for research, the wolves and dogs come from several genetic lines (wolves: 6, dogs: 5) suggesting that the results are not specific to certain family lines. The second limitation, retesting animals that already participated in a similar study, we actually consider a strength: The fact that we still found no differences between wolves and dogs in their OSM abilities despite using a very different experimental set-up suggests that the previous results were not driven by some methodological aspects of the experimental set-up.

In conclusion, our results corroborate our hypothesis that both wolves and dogs possess OSM and that wolves and dogs differ in other traits such as persistency and motivation to try to solve food-related challenges (see also [34, 51, 52]).

## Supporting information

S1 Data (CSV)

S2 Data (XLSX)

S3 Data (CSV)

S4 Data (R)

S5 Data (CSV)
